# Business analysis and market potential of drug screening technology for preventive and personalized therapy

**DOI:** 10.1186/1878-5085-5-S1-A105

**Published:** 2014-02-11

**Authors:** Igor V Volchek, Philip D Cotter, Andrei S Petrov, Mathew W Moore

**Affiliations:** 1 DiscoveryMed Ltd, St. Petersburg, Russia; 2 ResearchDx, Pacific Diagnostic Clinical Laboratory, Irvine, CA, US

## 

We have taken the business analysis to evaluate US market potential of new technology for preventive and personalized therapy (PT) of human diseases – Method for screening drug preparations (Method) [1] and it impact on the public health. Six sexually transmitted diseases (HCV, HBV, HPV, HSV, HIV and genital chlamidiosis), influenza, other acute respiratory diseases (ARD) and cancer [2] were used for the analysis. PT can allow to save up to $17.5 – 19.25 billion for Medicare/Medicaid for US hepatitis C market. The cost of the treatment of one patient with melanoma stage II - III can decrease 3-fold (saved $3,950 per patient per year) while the efficacy have to increase 2 – 3-fold. At cancer forms with no effective and safe adjuvant therapy PT can decrease the rate of metastatic stage which requires high-cost cytostatic therapy. PT with antiretrovirals and cytostatics can allow reduction of high expense of hospital stay for the patients with AIDS and metastatic cancer due to higher efficacy. PT will decrease the rate of non-effective treatment and number of relapses of HPV-infection, genital herpes and urogenital chlamidyosis as well as the number of new cases due to less spreading via sexual contacts. Preventive PT of HPV-infection with unfavorable HPV types will lead to less number of cases with cervical neoplasia. Preventive PT in risk group of 1% cases of influenza (950,000 cases) will give the ability for the insurance companies to save money for the treatment of 2.8 – 3.4 mln episodes of influenza and ARD. At the same time the number of restricted-activity days will decrease for 10 – 12 mln, bed days – for 5 – 7 mln and work-loss days due to flu and other ARD – for 2 – 2.5 mln.

The Market potential data of Method are summarized in the table below.

**Table 1. US estimated market potential of the Method for Screening Drug Preparations:** 

Diseases	Treatment Annual Income/ ($ mln)	Treatment market/5 Years ($ mln)	Prevention market ($ mln)
1. Hepatitis C	125 – 300	625 – 1,500	

2. HPV infection	125 – 250	625 – 1,250	900

3. AIDS/HIV	125 – 210	625 – 1,050	

4. Urogenital chlamydiosis	35	175	

5. Genital herpes	25	125	3,000

6. Hepatitis B	20	100	

**STD (subtotal)**	**455 – 840**	**2,275 – 4,200**	**3,900**

7. Influenza & ARD			450 (5 years)

8. Cancer	300	1,500	???

**Total**	**755 – 1,140**	**3,775 – 5,700**	**4,350**

Other perspective directions of PT using proposed Method: atherosclerosis, diabetes, endometriosis, chronic fatigue syndrome; CMV-, EBV-, *HelicobacterPylori* infections, sepsis and tuberculosis.
